# Report of Four Cases with Equestrian Injury: Therapeutic Approach and Outcome

**DOI:** 10.1155/2018/8283179

**Published:** 2018-06-27

**Authors:** Kiyohiro Oshima, Masato Murata, Makoto Aoki, Jun Nakajima, Yusuke Sawada, Yuta Isshiki, Yumi Ichikawa, Shuichi Hagiwara

**Affiliations:** Department of Emergency Medicine, Gunma University Graduate School of Medicine, Japan

## Abstract

Equestrianism is associated with a risk of severe trauma due to falls and/or direct injury from the horse, depending on the mechanism of injury. This article presents four cases of equestrian injury treated in Gunma University Hospital: Case 1: hepatic injury (fall and kick by the horse); Case 2: left hemopneumothorax and pulmonary contusion with multiple rib fractures (fall and trampling by the horse); Case 3: lumbar compression fracture (fall); and Case 4: scrotum injury (horse bite). Equestrian injuries may be high-energy traumas. Therefore, adhering to relevant primary care guidelines may prevent mortality by trauma.

## 1. Introduction

Equestrianism is associated with a risk of severe trauma due to falls and/or direct injury from the horse, depending on the mechanism of injury. However, horse riding is not a common sport in Asian countries such as Hong Kong [1] and Japan [2]; therefore, equestrian injuries are rare in this region. This article presents four cases of equestrian injury treated in Gunma University Hospital in Japan. Informed consent was obtained from all four patients.

## 2. Presentation of Cases

### 2.1. Case 1

**Figure 1:**
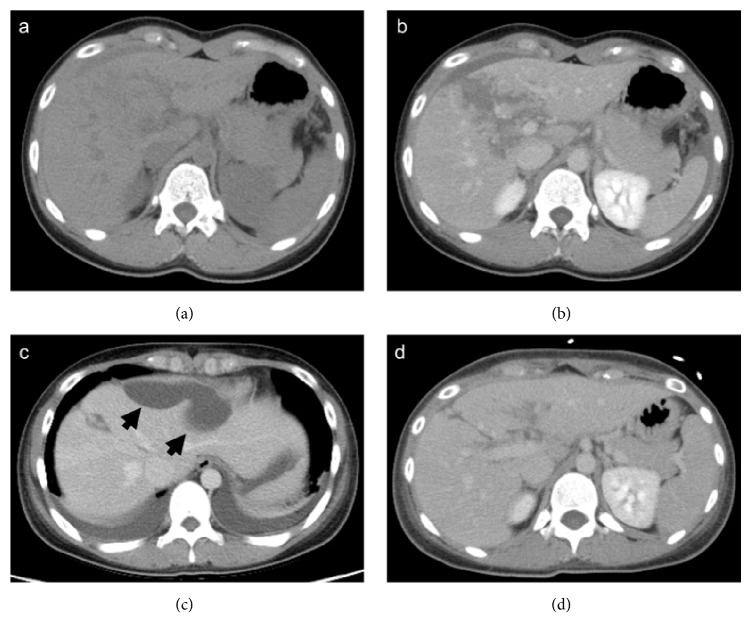
(a) Computed tomography (CT) performed on arrival at the hospital showing hepatic injury. (b) Enhanced CT performed on arrival at the hospital showing hepatic injury. (c) Enhanced CT performed on day 17 of hospitalization showing biloma (black arrows). (d) Enhanced CT performed on day 34 of hospitalization showing that the biloma had almost disappeared.

A 20-year-old female fell and was kicked several times by the horse on the thoracoabdominal area. The patient was transferred to the emergency room (ER). Vital signs included blood pressure: 122/82 mmHg; heart rate: 72 beats per minute (bpm); body temperature: 36.3°C; respiratory rate: 18/min; and SpO2: 100% (ambient air). The patient complained of epigastralgia and tenderness was observed at the same site. Focused assessment with sonography for trauma (FAST) was positive at the Morison's pouch, perisplenic and pericystic regions. Laboratory data revealed elevated levels of hepatic enzymes (aspartate aminotransferase: 174 U/l; alanine aminotransferase: 149 U/l). Enhanced abdominal computed tomography (CT) showed a hepatic injury (abbreviated injury scale [AIS]: 4; revised trauma score [RTS]: 7.8408; probability of survival [Ps]: 0.94702; Figures 1(a)-1(b)). Enhanced CT did not show obvious extravasation and the patient's general condition was stable. Thus, nonoperative management (NOM) was selected. Transcutaneous drainage of the biloma was performed (Figure 1(c)) and the drainage tube was removed (Figure 1(d)) on days 17 and 34 of hospitalization, respectively. The clinical course was stable and the patient was discharged on day 37 of hospitalization. Forty months after injury the patient has fully recovered.

### 2.2. Case 2

**Figure 2:**
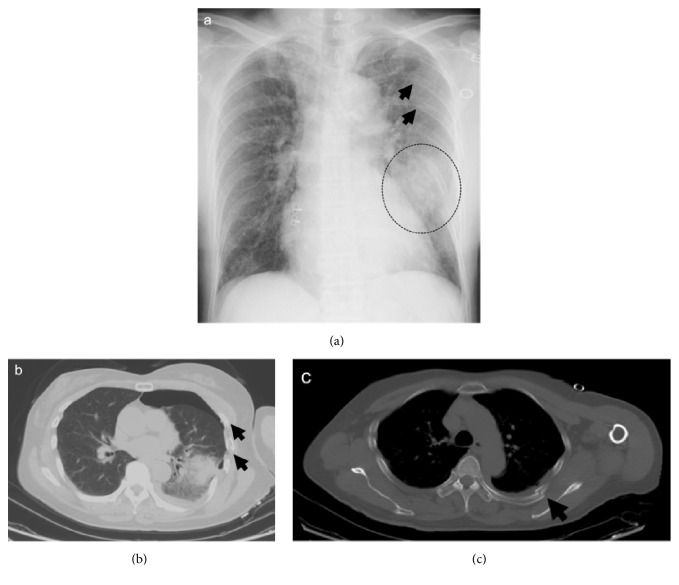
(a) Chest roentgenogram showing pulmonary contusion (dotted circle) with rib fractures (black arrows). (b and c) Chest CT showing left hemopneumothorax and pulmonary contusion with multiple rib fractures (black arrows).

A 60-year-old female fell and was trampled on the left side of the chest by the horse. The patient was transferred to the ER. Vital signs included blood pressure: 156/116 mmHg; heart rate: 113 bpm; body temperature: 36.5°C; respiratory rate: 30/min; and SpO2: 96% (ambient air). An abrasion on the left chest without flail chest was observed. Chest roentgenography and CT showed left hemopneumothorax and pulmonary contusion with multiple rib fractures (AIS: 3; RTS: 7.55; Ps: 0.95590; Figures 2(a)-2(c)). A chest tube was placed to the left chest and NOM was selected for the rib fractures due to the little deviation. The patient's respiratory condition was stable, and endotracheal intubation and mechanical ventilation were not required during the course of treatment. The chest tube was removed on day 6 and the patient was discharged on day 8 of hospitalization. Twenty-seven months after injury the patient has fully recovered.

### 2.3. Case 3

**Figure 3:**
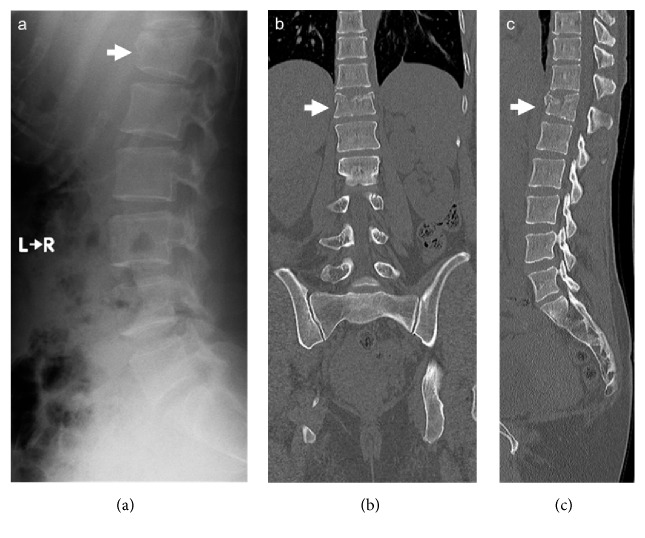
(a) Roentgenogram (a) and CT (b and c) showing compression fracture of Th12 (white arrows).

A 30-year-old female fell and was injured in the lumbar area. The patient was transferred to the ER. Vital signs included blood pressure: 159/91 mmHg; heart rate: 77 bpm; body temperature: 36.5°C; respiratory rate: 12/min; and SpO2: 100% (ambient air). The patient complained of lumbago. Roentgenography and CT showed a compression fracture of Th12 (AIS: 3; RTS: 7.8408; Ps: 0.99416; Figures 3(a)-3(c)). Conservative therapy using a medical corset was advised, and the patient was discharged on day 17 of hospitalization. Thirty-seven months after injury the patient has fully recovered.

### 2.4. Case 4

A 50-year-old male fell and was bitten on the crotch by the horse. The patient was transferred to the ER. Vital signs included blood pressure: 161/118 mmHg; heart rate: 71 bpm; and body temperature: 36.3°C. There was a 5-cm laceration on the right scrotum (AIS: 1; RTS: 7.8408; Ps: 0.99700). The tunica vaginalis of the right testis was macroscopically intact, while ultrasonography did not reveal abnormal findings in the right testis. The laceration was sutured after cleansing with normal saline under local anesthesia. The clinical course was uneventful and the patient was discharged from the hospital the following day.

## 3. Discussion

Equestrian injury is common with over 100,000 cases reported in the United States of America (USA) annually [3]. The incidence of equestrian injuries has also been well researched in the United Kingdom and Australia [1]. Paix reported that in South Australia (1990-1998) equestrian injury occurred at a rate of less than 1/1,000 [4]. Carmichael et al. [5] reported that most injuries occurred due to falling off while riding (54%) or being kicked by the horse (22%), resulting in extremity fractures (33%) or head injury (27%). In addition, mounted equestrians more commonly suffer injuries to the chest and lower extremity, whereas unmounted equestrians suffer injuries to the face and abdomen more frequently. The incidence of head trauma was similar between mounted and unmounted equestrians. Moreover, the investigators highlighted that reduction in head trauma coincides with reduction of mortality due to equestrian injury. Balakrishnan et al. [6] evaluated 20 patients with liver trauma caused by equestrian injury, reporting that laparotomy is rarely warranted and conservative management in the form of close observation may be sufficient. In the present cases, conservative management was also successfully performed.

Gunma University Hospital is the nearest emergency medical center to Gunma equestrian park (<10 km) and has an important role in treating patients with equestrian injuries. In Japan, the present medical care for trauma is performed according to the Japan Advanced Trauma Evaluation and Care (JATEC) guideline of the Japanese Association for the Surgery of Trauma and the Japanese Association for Acute Medicine. The JATEC guideline was based on the Advanced Trauma Life Support® (ATLS®) program of the USA and was adapted to the conditions of Japan. The guidelines recommend to initially recognize the physiological abnormalities in trauma patients and perform resuscitation if necessary (primary survey) and subsequently evaluate anatomical abnormalities (secondary survey).

Equestrian injuries may be high-energy traumas. Therefore, adhering to relevant primary care guidelines may prevent mortality by trauma.

## References

[1] Yim V. W. T., Yeung J. H. H., Mak P. S. K., Graham C. A., Lai P. B. S., Rainer T. H. (2007). Five year analysis of Jockey Club horse-related injuries presenting to a trauma centre in Hong Kong. *Injury*.

[2] Oode Y., Maruyama T., Kimura M., Fukunaga T., Omori K., Yanagawa Y. (2016). Horse kick injury mimicking a handle bar injury or a hidden speared injury. *Acute Medicine & Surgery*.

[3] McCrory P., Turner M. (2005). Equestrian injuries. *Medicine and Sport Science*.

[4] Paix B. R., Whitlock M. (1999). Rider injury rates and emergency medical services at equestrian events. *British Journal of Sports Medicine*.

[5] Carmichael S. P., Davenport D. L., Kearney P. A., Bernard A. C. (2014). On and off the horse: Mechanisms and patterns of injury in mounted and unmounted equestrians. *Injury*.

[6] Balakrishnan A., Abbadi R., Oakland K. (2014). Outcomes following liver trauma in equestrian accidents. *Journal of Trauma Management & Outcomes*.

